# El Coléra en la República Argentina

**Published:** 1898-03

**Authors:** 


**El Colera en la Republica Argentina.** Por el Doctor José Penna. Pp. xv., 361. Buenos Aires: Jacobo Peuser. 1897.

This is an elaborate account of an invasion of the Argentine Republic by cholera during forty years—from 1856 to 1895. It originally broke out among the soldiers of a military expedition directed against Brazil in the first year mentioned, and. when the army returned home, they carried the disease to the inhabitants of Buenos Aires, the capital, and there it remained endemic until 1867, when the smouldering fire blazed forth, and the cholera was responsible for 3000 deaths in the city and the surrounding country in the early months of that year; thence it spread to the neighbouring States of Uruguay and Paraguay, affecting severely their respective capitals of Monte Video and Asuncion, and also the districts of Corrientes, Santa Fé, Santiago, and Cordova. In the epidemic of 1893 the mortality from cholera reached 1000, and the ratio was 45 per cent. of those attacked. But the worst outbreak was in 1886, when 11,000 succumbed to the disease in the Argentine and adjacent Republics, and the proportion of deaths to those attacked attained the high figure of 75 per cent. From that time the epidemics became less frequent and the fatalities were not so numerous, owing to the careful sanitary measures taken by the Government, prompted by a better knowledge of hygiene, until in 1895 the disease disappeared altogether.

